# Anaphylatoxin Receptors C3aR and C5aR1 Are Important Factors That Influence the Impact of Ethanol on the Adipose Secretome

**DOI:** 10.3389/fimmu.2018.02133

**Published:** 2018-09-20

**Authors:** Rebecca L. McCullough, Megan R. McMullen, Kyle L. Poulsen, Adam Kim, M. Edward Medof, Laura E. Nagy

**Affiliations:** ^1^Department of Inflammation and Immunity, Center for Liver Disease Research, Lerner Research Institute, Cleveland Clinic, Cleveland, OH, United States; ^2^Institute of Pathology, Case Western Reserve University, Cleveland, OH, United States; ^3^Department of Gastroenterology and Hepatology, Cleveland Clinic, Cleveland, OH, United States

**Keywords:** adipose, inflammation, secretome, complement, anaphylatoxin, alcoholic liver disease, extracellular vesicles

## Abstract

**Background and aims:** Chronic ethanol exposure results in inflammation in adipose tissue; this response is associated with activation of complement as well as the development of alcohol-related liver disease (ALD). Adipose communicates with other organs, including liver, via the release of soluble mediators, such as adipokines and cytokines, characterized as the “adipose secretome.” Here we investigated the role of the anaphaylatoxin receptors C3aR and C5aR1 in the development of adipose tissue inflammation and regulation of the adipose secretome in murine ALD (mALD).

**Methods:** Wild-type C57BL/6 (WT), *C3aR*^−/−^, and *C5aR1*^−/−^ mice were fed Lieber-DeCarli ethanol diet for 25 days (6% v/v, 32% kcal) or isocaloric control diets; indicators of inflammation and injury were assessed in gonadal adipose tissue. The adipose secretome was characterized in isolated adipocytes and stromal vascular cells.

**Results:** Ethanol feeding increased the expression of adipokines, chemokines and leukocyte markers in gonadal adipose tissue from WT mice; *C3aR*^−/−^ were partially protected while *C5aR1*^−/−^ mice were completely protected. In contrast, induction of CYP2E1 and accumulation of TUNEL-positive cells in adipose in response to ethanol feeding was independent of genotype. Bone marrow chimeras, generated with WT and *C5aR1*^−/−^ mice, revealed C5aR1 expression on non-myeloid cells, likely to be adipocytes, contributed to ethanol-induced adipose inflammation. Chronic ethanol feeding regulated both the quantity and distribution of adipokines secreted from adipocytes in a C5aR1-dependent mechanism. In WT mice, chronic ethanol feeding induced a predominant release of pro-inflammatory adipokines from adipocytes, while the adipose secretome from *C5aR1*^−/−^ mice was characterized by an anti-inflammatory/protective profile. Further, the cargo of adipocyte-derived extracellular vesicles (EVs) was distinct from the soluble secretome; in WT EVs, ethanol increased the abundance of pro-inflammatory mediators while EV cargo from *C5aR1*^−/−^ adipocytes contained a greater diversity and more robust expression of adipokines.

**Conclusions:** C3aR and C5aR1 are potent regulators of ethanol-induced adipose inflammation in mALD. C5aR1 modulated the impact of chronic ethanol on the content of the adipose secretome, as well as influencing the cargo of an extensive array of adipokines from adipocyte-derived EVs. Taken together, our data demonstrate that C5aR1 contributes to ethanol-mediated changes in the adipose secretome, likely contributing to intra-organ injury in ALD.

## Introduction

Alcohol abuse is a global health burden, contributing significantly to preventable chronic liver disease in the western world. Alcohol injures the liver directly via its toxicity, but also indirectly by contributing to extrahepatic organ damage, including intestine, skeletal muscle, kidney, and adipose tissue (1, 2). While the mechanisms are less well-understood, there is increasing recognition that extrahepatic alcohol toxicity adds to the multiple “hits” that impact the progression of alcohol-related liver disease (ALD) (3). Limited therapeutic options exist for patients with ALD; a better understanding of organ-organ crosstalk in ALD will likely reveal potential therapeutic targets for individuals with ALD.

Adipose tissue is an important energy storage unit, but it also participates in regulation of whole-body metabolism via its endocrine functions, secretion of adipokines and regulation of glucose and energy homeostasis (4). Adipose tissue is a significant target of alcohol toxicity; ethanol perturbs lipid metabolism, induces oxidative stress via CYP2E1, dysregulates adipokine secretion and induces adipose tissue inflammation (1, 4–7). While the innate immune system is known to play a role, the mechanisms contributing to ethanol-induced adipose tissue inflammation are not well-understood.

Complement, an integral component of the innate immune system, is central in orchestrating systemic immune responses. In addition to acting as an immune surveillance mechanism, a growing body of evidence suggests complement is a key modulator of metabolic diseases, including non-alcoholic and alcoholic liver disease. Complement is activated via the classical, lectin and alternative pathways, converging at complement component 3 (C3) (8). Initiation of the terminal pathway leads to C3 and complement component 5 (C5) cleavage, generating the potent anaphylatoxins C3a and C5a, respectively. In addition to their chemokine-like activity, C3a and C5a can activate cells via their cognate G-protein coupled receptors (GPCR), C3aR and C5aR1, to elicit a multitude of cellular responses, including the secretion of pro-inflammatory mediators. C5a and its degradation product C5a-des-Arg also bind to the non-GPCR C5a receptor-2 (C5aR2), however the physiologic role for C5aR2 is less known (9). Adipose is a major producer of complement proteins, including C3, components of the alternative pathway (factor B and D) and complement receptors (C3aR and C5aR1) (6, 10–13).

The inflammatory response in adipose tissue induced by ethanol is multifaceted, perpetuated in part by soluble mediators secreted by adipocytes and components of the innate immune system. These soluble mediators, including adipokines, cytokines, and chemokines, regulate metabolism in multiple tissues, including liver and comprise the adipose tissue secretome. Ethanol increases the secretion of pro-inflammatory cytokines (e.g., tumor necrosis factor alpha [TNFα] and interleukin-6 [IL-6]) and chemokines (e.g., monocyte chemoattractant protein 1 [MCP-1]); persistent activation of the innate immune system by alcohol contributes to immune cell recruitment and tissue injury. Indeed, dysregulated chemokine/cytokine/adipokine networks have been reported in humans and animals models of ALD (1, 5, 6). Collectively, all of these changes in adipose homeostasis are postulated to contribute to ALD pathogenesis; however, the mechanisms by which adipose communicates with liver in ALD are not well-understood.

Extracellular vesicles (EVs) are an understudied component of the adipose secretome. EVs are small (< 200 nm) membrane-bound vesicles that mediate paracrine communication and signaling in various diseases, including ALD (14, 15). EVs from adipose are implicated to have important roles in whole body metabolism and insulin resistance (16), but their role in ethanol-induced adipose inflammation is not known.

Complement activation products are increased in ALD patients and in murine ALD (mALD) (8, 17, 18). While the anaphylatoxin receptors have well-defined roles in modulating inflammation, less is known about their role in moderating adipose inflammation in mALD. Accumulation of cleavage products (C3b/iC3b/C3c) can be detected in adipose after ethanol feeding in mice (6); therefore, we tested the hypothesis that the anaphylatoxins, generated in response to ethanol feeding, contribute to non-resolving adipose inflammation and tissue injury in mALD. Here, utilizing mice deficient in *C3aR* and *C5aR1*, we have found an important role for anaphylatoxin receptors in regulating the expression of inflammatory factors in adipose tissue following chronic ethanol feeding in mice. Moreover, we find that C5aR1 on adipocytes contributes to ethanol-induced adipose inflammation via the secretion of adipokines. Importantly, C5aR1 differentially modulated EV cargo from isolated adipocytes isolated from ethanol-fed mice. Collectively, these data highlight the impact of both C3aR and C5aR1 on adipose inflammation and content of the adipose secretome in mALD; complement receptor-dependent changes in the secretome may provide a critical link to hepatic injury and progression of ALD.

## Materials and methods

### Animals

Female 8–10 weeks old C57BL/6 (WT), *C3aR*^−/−^, and *C5aR1*^−/−^ mice were used for all experiments. WT mice were purchased from Jackson Laboratories (Bar Harbor, ME). Complement receptor-deficient mice were backcrossed to a C57BL/6 background and were bred in-house at the Cleveland Clinic (19, 20). Genotyping was routinely performed in complement KO mice using DNA extracted from tails with a DNeasy Blood & Tissue Kit per the manufacturer's instructions (Qiagen, Germantown, MD); primer sequences used for genotyping were: *C3aR*^−/−^: Common Forward 5′-AGC CAT TCT AGG GGC GTA TT-3′, WT Reverse: 5′-CAT GGT TTG GGG TTA TTT CG-3′, Mutant Reverse: 5′-TGG ATG TGG AAT GTG TGC GAG-3′; *C5aR1*^−/−^: WT Forward: 5′-GGT CTC TCC CCA GCA TCA TA-3′, Mutant Forward: 5′-GCC AGA GGC CAC TTG TGT AG-3′, Common Reverse: 5′-GGC AAC GTA GCC AAG AAA AA-3′.

### Chronic ethanol feeding

All procedures using animals were approved by the Cleveland Clinic Institutional Animal Care and Use Committee. Female mice (WT, *C3aR*^−/−^, and *C5aR1*^−/−^) were housed in standard microisolator cages (2 animals/cage). Lieber-DeCarli high-fat ethanol and control diets were purchased from Dyets (Bethlehem, PA; Cat#710260). Age- and weight-matched animals were randomized into ethanol-fed and pair-fed groups, adapted to control liquid diet for 2 days and fed increasing concentrations of ethanol up to 6% v/v (32% kcal) for 25d as previously described (21). Ethanol-fed mice were allowed *ad libitum* access to liquid diet. Control mice were pair-fed a diet that received isocalorically-substituted maltose dextrins for ethanol. At the termination of the study, mice were anesthetized, blood was taken into non-heparinized syringes from the posterior vena cava, and tissues excised. Blood was transferred into EDTA-containing Microtainer tubes (BD Biosciences, Franklin Lakes, NJ) for isolation of plasma and stored at −80°C until further analysis. Portions of the gonadal adipose tissue were either used for primary cell isolations or fixed in formalin for histology, flash frozen in liquid nitrogen or put in RNA later and stored at −80°C until further analysis.

### Generation of C5aR1 chimeras

Bone marrow transplants were performed using WT and *C5aR1*^−/−^ mice as previously described (22). Briefly, following lethal irradiation, WT and *C5aR1*^−/−^ recipients received donor marrow via I.V. tail vein injection from *C5aR1*^−/−^ and WT mice, respectively. After 5 weeks of recovery, chimeras were dosed I.V. with clodronate-containing liposomes (Encapsula NanoSciences, Nashville, TN; SKU#8909) to deplete tissue resident macrophages. Seven days later, chimeric mice started the chronic ethanol feeding. In a separate experiment, WT mice were dosed I.V. with clodronate liposomes; 48 h post-clodronate, tissues were excised and analyzed for F4/80 mRNA by qRT-PCR to validate macrophage depletion. Clodronate depleted F4/80 mRNA expression in gonadal adipose by ~80% (data not shown).

### Primary cell isolations from gonadal adipose tissue

Adipocytes and stromal vascular cells (SVCs) were collected from gonadal fat pads of pair-fed or ethanol-fed mice following digestion using Hanks(+) media containing 100 mM HEPES, pH 7.4, 1% RIA grade bovine serum albumin, Type II collagenase (1 mg/g fat) and 50 nM Adenosine (5). Digested tissue was passed through 250 μM course mesh, infranatant was collected below and floating adipocytes and stromal vascular cells (SVCs) were separated from the infranatant by centrifugation (300 × g). Following red cell lysis, SVCs were collected via centrifugation and used for secretion assays. Adipocytes (1 × 10^5^ cells) or SVCs (1.25 × 10^5^ cells) were diluted in PBS and transferred to 1.5 mL Eppendorf tubes. Spontaneous secretion of soluble factors were measured up to 120 min after the isolation by ELISA. Pooled adipocyte supernatants (*n* = 6–8/group) from the 60 min time point were used for an Adipokine Array (R&D Systems, Minneapolis, MN).

### Enzyme-linked immunosorbant assays

The following ELISAs were performed in supernatants from isolated adipocytes and SVCs according to the manufacturer's protocol: MCP-1 (Mouse MCP-1 ELISA MAX, Biolegend, San Diego, CA), TNF-α (Mouse TNF-α ELISA MAX, Biolegend, San Diego, CA), Chemerin (Mouse Chemerin DuoSet ELISA, R&D Systems, Minneapolis, MN), lipocalin-2 (LCN2) (Mouse LCN2/NGAL DuoSet ELISA, R&D Systems, Minneapolis, MN) and soluble receptor for advanced glycation end products (sRAGE) (Mouse sRAGE DuoSet ELISA, R&D Systems, Minneapolis, MN).

### Isolation of extracellular vesicles (EVs)

Secreted EVs from isolated adipocytes (120 min timepoint) were enriched using PEG 8000-based methods as described previously (23). Samples from 6 to 8 mice/group were pooled for each representative group. Briefly, samples were centrifuged at 3,000 × g for 5 min to remove cellular debris. Supernatants were then passed through 0.22 μM Costar Spin-X cellulose acetate filter tubes to remove apoptotic bodies. 40% PEG 8000 was added to pre-cleared supernatants (final concentration of 8% PEG 8000) and rotated end-over-end overnight at 4°C. Following precipitation, EVs were pelleted via centrifugation at 5,000 × g for 30 min. EVs were lysed with 1%TritonX-100 in PBS for 10 min on ice; 4 × lysis buffer (20 mM HEPES, pH7.4; 150 mM NaCl; 1.5 mM MgCl_2_ and 2 mM EGTA) containing protease and phosphatase inhibitors (cat#A32959; ThermoFisher, Grand Island, NY) was added for an additional 30 min on ice. EV protein content was measured via BCA protein assay and 10 ug protein was used to measure EV content via an Adipokine Array per the manufacturer's instructions (R&D Systems, Minneapolis, MN). Isolation of EVs was verified via Western blotting using EV specific markers ALIX, TSG101, and HSC70 (24, 25).

### RNA isolation and quantitative real-time polymerase chain reaction (qRT-PCR)

RNA was isolated from gonadal adipose tissue stored in RNAlater using an RNeasy Lipid Tissue Mini kit per the manufacturer's instructions (Qiagen, Germantown, MD). 1 μg of adipose RNA was reverse transcribed and analyzed with PowerSYBR qRT-PCR kits (Applied Biosystems, Foster City, CA) on a QuantStudio5 analyzer (Applied Biosystems, Foster City, CA). Relative messenger RNA (mRNA) expression was determined using gene-specific primers listed in Table 1. Statistical analyses were performed on the ΔCt values (average Ct of gene of interest – average Ct of 18S) (26).

**Table 1:** Gene-specific primers used for qRT-PCR.

**Gene**	**Forward (5^′^-3^′^)**	**Reverse (5^′^-3^′^)**
Tumor necrosis factor alpha (*TNFα*)	CCC TCA CAC TCA GAT CAT CTT CT	GCT ACG ACG TGG GCT ACA G
Interleukin-6 (*IL-6*)	TAG TCC TTC CTA CCC CAA TTT CC	TTG GTC CTT AGC CAC TCC TTC
Interleukin-1 beta (*IL-1β*)	ATG GCA ACT GTT CCT GAA CTC AAC T	CAG GAC AGG TAT AGA TTC TTT CCT TT
Prostaglandin E Synthase 2 (PTGES2)	CCT CGA CTT CCA CTC CCT G	TGA GGG CAC TAA TGA TGA CAG AG
Lipocalin 2 (LCN2)	ATG CAC AGG TAT CCT CAG GT	TGG CGA ACT GGT TGT AGT CC
Chemerin	GCC TCG CTA AAG CAA CAA ACC	TGG GTG TTT GTG GAA CTC CT
C3a Receptor (*C3aR*)	TCG ATG CTG ACA CCA ATT CAA	TCC CAA TAG ACA AGT GAG ACC AA
C5a Receptor 1 (*C5aR1*)	GTG GGT TTT GTG TTG CCT CT	TGA TAG GGC AGC CAG AAG AT
C5a Receptor 2 (*C5aR2*)	ACC ACC AGC GAG TAT TAT GAC T	GCT GCA TAC AGC ACA AGC A
C-X-C Motif Chemokine Ligand 1 (*CXCL1/KC*)	TGC ACC CAA ACC GAA GTC	GTC AGA AGC CAG CGT TCA CC
C-X-C Motif Chemokine Ligand 2 (*CXCL2/MIP2α*)	GCG CCC AGA CAG AAG TCA TAG	AGC CTT GCC TTT GTT CAG TAT C
C-C Motif Chemokine Ligand 2 (*CCL2/MCP-1*)	AGG TCC CTG TCA TGC TTC TG	TCT GGA CCC ATT CCT TCT TG
C-C Motif Chemokine Ligand 3 (*CCL3/MIP1α*)	TTC TCT GTA CCA TGA CAC TCT GC	CGT GGA ATC TTC CGG CTG TAG
C-C Motif Chemokine Ligand 5 (*CCL5/RANTES*)	GCT GCT TTG CCT ACC TCT CC	TCG AGT GAC AAA CAC GAC TGC
Integrin Subunit Alpha X (*ITGAX/CD11c*)	CTG GAT AGC CTT TCT TCT GCT G	GCA CAC TGT GTC CGA ACT CA
Integrin Subunit Alpha M (*CD11b*)	ATG GAC GCT GAT GGC AAT ACC	TCC CCA TTC ACG TCT CCC A
Adhesion G protein-coupled receptor E1 (*F4/80*)	CCC CAG TGT CCT TAC AGA GTG	GTG CCC AGA GTG GAT GTC T
Lymphocyte antigen 6C2 (*Ly6C*)	GCA GTG CTA CGA GTG CTA TGG	ACT GAC GGG TCT TTA GTT TCC TT
Lymphocyte antigen 6G6e (*Ly6G*)	TGC GTT GCT CTG GAG ATA GA	CAG AGT AGT GGG GCA GAT GG

### Immunohistochemistry and terminal deoxynucleotidyl transferase-mediated dUTP nick end labeling (TUNEL) staining

For histological analysis, formalin-fixed tissues were paraffin-embedded, sectioned and stained with hematoxylin and eosin. Paraffin embedded gonadal adipose sections were deparaffinized and stained with an antibody against TNFα (Fitzgerald Industries International, Acton, MA; cat# 70R-TR008) (22, 27). Images were captured on an upright microscope (Olympus Corp, Center Valley, PA). At least three images were acquired per tissue section and semi-quantification of positive staining was performed using ImagePro Plus software (Media Cybernetics, Silver Spring, MD). No specific immunostaining was seen in sections incubated with PBS rather than the primary antibody (data not shown). Apoptotic DNA fragmentation was detected by TUNEL using the ApopTag Peroxidase *in situ* apoptosis detection kit (Millipore, Temecula, CA) on paraffin embedded gonadal adipose sections according to the manufacturer's protocol. Percent TUNEL-positive nuclei of total nuclei was determined by counting TUNEL-positive cells and hematoxylin staining from three different fields per slide using ImagePro Plus software. Samples are coded at time of collection for a blinded analysis.

### Immunoblotting

Protein lysates were made from frozen adipose in lysis buffer containing: 0.5% Triton X-100, 20 mM HEPES (pH 7.4), 150 mM MgCl_2_, 2 mM EGTA, 10 mM NaF, 1 mM PMSF, 1 mM Na_3_(VO_3_)_4_, 12.5 mM βGP, 2 mM DTT, and protease inhibitor cocktail (Roche Diagnostics, Indianapolis, IN, Cat# 14826500). Protein concentrations were determined by the DC Protein Assay (Biorad, Hercules, CA) and samples were denatured at 95°C in Laemmli buffer. Samples were separated on SDS-PAGE gels, transferred to polyvinylidene fluoride membranes with a semi-dry transfer apparatus (Biorad, Hercules, CA), and blocked in 3% bovine serum albumin. Membranes were then probed with CYP2E1 (EMD Millipore, Darmstadt, Germany), ALIX (Cell Signaling, Danvers MA), TSG101 and HSC70 (Santa Cruz Biotechnology, Santa Cruz, CA). Horseradish peroxidase-conjugated secondary antibodies (Santa Cruz Biotechnology, Santa Cruz, CA) were applied, and membranes were developed using Immobilon Western Developing reagents (Millipore, Temecula, CA). Chemiluminescence was visualized using a CL-XPosure Film (ThermoFisher).

### Generation of secretome heatmaps

Data (secreted adipokines and EV cargo) were imported, log transformed and dendrograms generated using RStudio software (28). Hierarchical clustering analysis was performed and heatmaps were visualized using ggplot2 (29).

### Statistical analysis

Values reported are means ± SEM (*n* ≥ 4–8 for pair-fed, *n* ≥ 6–10 for ethanol-fed mice. The data were analyzed by general linear models procedure followed by least square means analysis of differences between groups; data were tested for normality using the Shapiro-Wilk test (SAS; Carey, NC). Data were log transformed to obtain a normal distribution, if necessary.

## Results

### Anaphylatoxin receptors C3aR and C5aR1 influence a broad array of adipose-derived inflammatory factors in ethanol-fed mice

**Figure 1:**
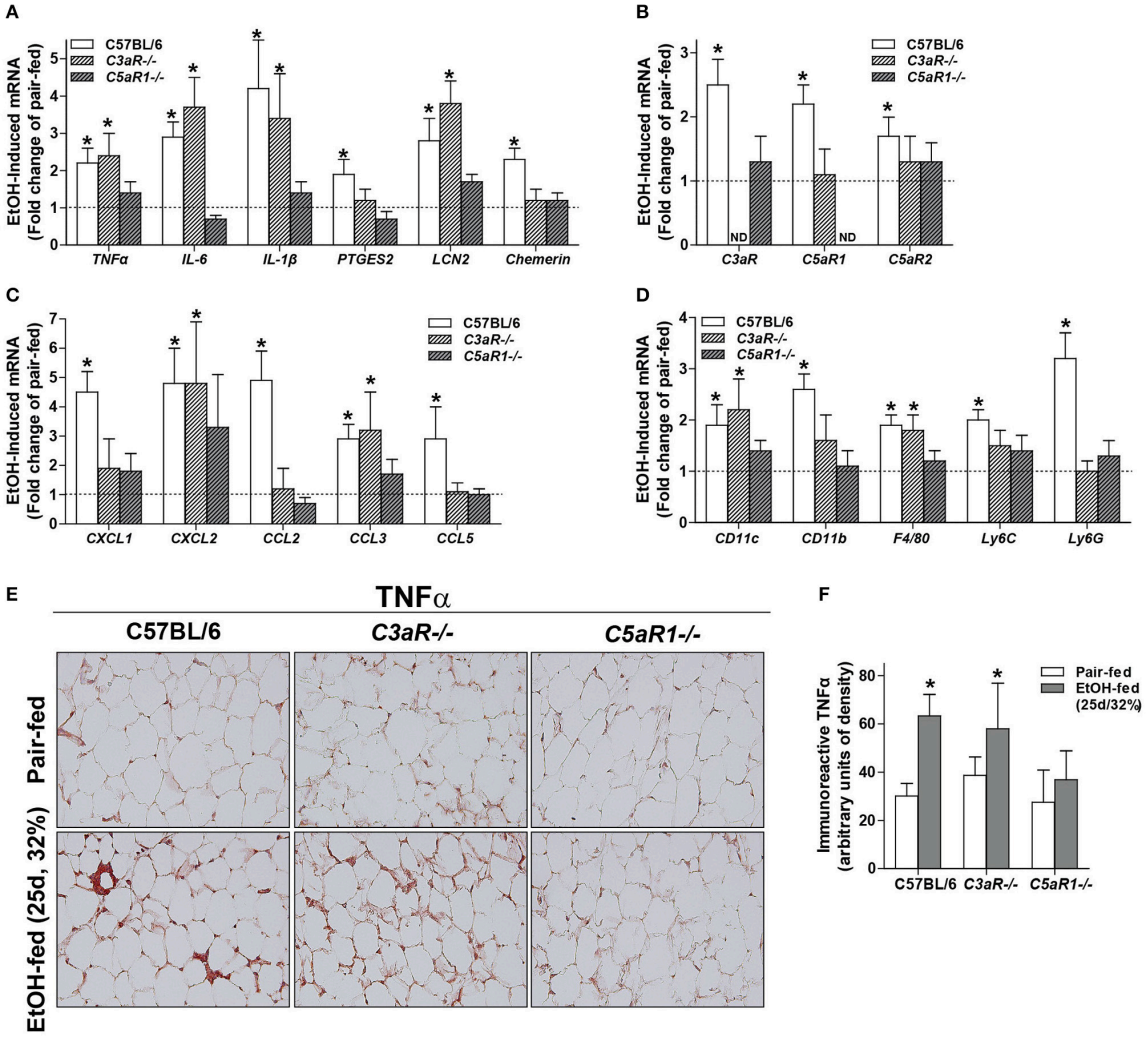
Chronic ethanol feeding increases the expression of inflammatory factors in WT and *C3aR*^−/−^ mice. WT, *C3aR*^−/−^, and *C5aR1*^−/−^ mice were allowed free access to ethanol (32%, d25) or pair-fed control diets. Expression of mRNA for **(A)** adipokines, cytokines, **(B)** complement receptors, **(C)** chemokines, and **(D)** leukocyte markers in gonadal adipose tissue. Values are expressed as the fold increase over genotype pair-fed controls. Values represent means ± SEM, values with ^*^were significantly different from pair-fed controls within genotype (*P*<*0.05*), *n* = 4–8 pair-fed, *n* = 6–12 EtOH-fed mice. **(E)** Paraffin-embedded gonadal adipose tissue was stained for TNFα. Nuclei were counterstained with hematoxlin. All images were acquired using a 20 × objective. **(F)** TNFα-stained areas were quantified from at least three images per slide using Image-Pro Plus software. Values represent means ± SEM, values with * were significantly different from pair-fed controls within genotype (*P*<*0.05*). *n* = 3–7 pair-fed and 4–12 EtOH-fed mice.

Adipose tissue is highly sensitive to alcohol; ethanol activates cellular components of the innate immune system to secrete cytokines/chemokines. Because anaphylatoxins are known to elicit pro-inflammatory actions on various immune cells, we tested the hypothesis that mice deficient in the anaphylatoxin receptors C3aR and C5aR1 would be protected from ethanol-induced adipose inflammation. In gonadal adipose tissue, markers characteristic of adipose inflammation, including *TNF*α, *IL-6, IL-1*β, *PTGES2, LCN2*, and *Chemerin* mRNA were increased in ethanol-fed WT, but not *C5aR1*^−/−^ mice compared to pair-fed controls (Figure 1A). Ethanol-fed *C3aR*^−/−^ mice were partially protected from increases in *PTGES2* and *Chemerin*, but not *TNF*α, *IL-6, IL-1*β, and *LCN2* mRNA. Ethanol feeding increased the mRNA expression of the analyphylatoxin receptors *C3aR, C5aR1* and *C5aR2* in WT mice, which was prevented in *C3aR*^−/−^ and *C5aR1*^−/−^ mice compared to pair-fed controls (Figure 1B).

C-X-C and C-C chemokines are key mediators of inflammation that lead to the infiltration of neutrophils and macrophages into tissues. Ethanol feeding increased the expression of *CXCL1* (*KC*), *CXCL2* (*MIP2*), *CCL2* (*MCP-1*), *CCL3* (*MIP-1*α), and *CCL5* (*RANTES*) in the gonadal adipose tissue of WT, but not *C5aR1*^−/−^ mice (Figure 1C). Ethanol feeding increased *CXCL2* and *CCL3*, but not *CXCL1, CCL2*, and *CCL5* mRNA in adipose from *C3aR*^−/−^ mice (Figure 1C). Ethanol feeding increased the expression of *CD11c, CD11b, F4/80, Ly6C*, and *Ly6G* mRNA in WT, but not *C5aR1*^−/−^ mice. Consistent with the partial protection observed with chemokine expression, ethanol feeding increased the expression of *CD11c, F4/80, Ly6G*, but not *CD11b* and *Ly6C* mRNA in adipose from *C3aR*^−/−^ mice (Figure 1D).

In WT and *C3aR*^−/−^, but not *C5aR1*^−/−^ mice, ethanol feeding increased the expression of TNFα in gonadal adipose tissue, with localized staining within crown-like structures (Figures 1E,F). Taken together, these data demonstrate that while C3aR partially contributes, C5aR1 is a more potent regulator of ethanol-induced adipose inflammation in mALD.

### Ethanol-fed *C3aR*^−/−^ and *C5aR1*^−/−^ mice have increased CYP2E1 expression and apoptotic cells in gonadal adipose tissue

**Figure 2:**
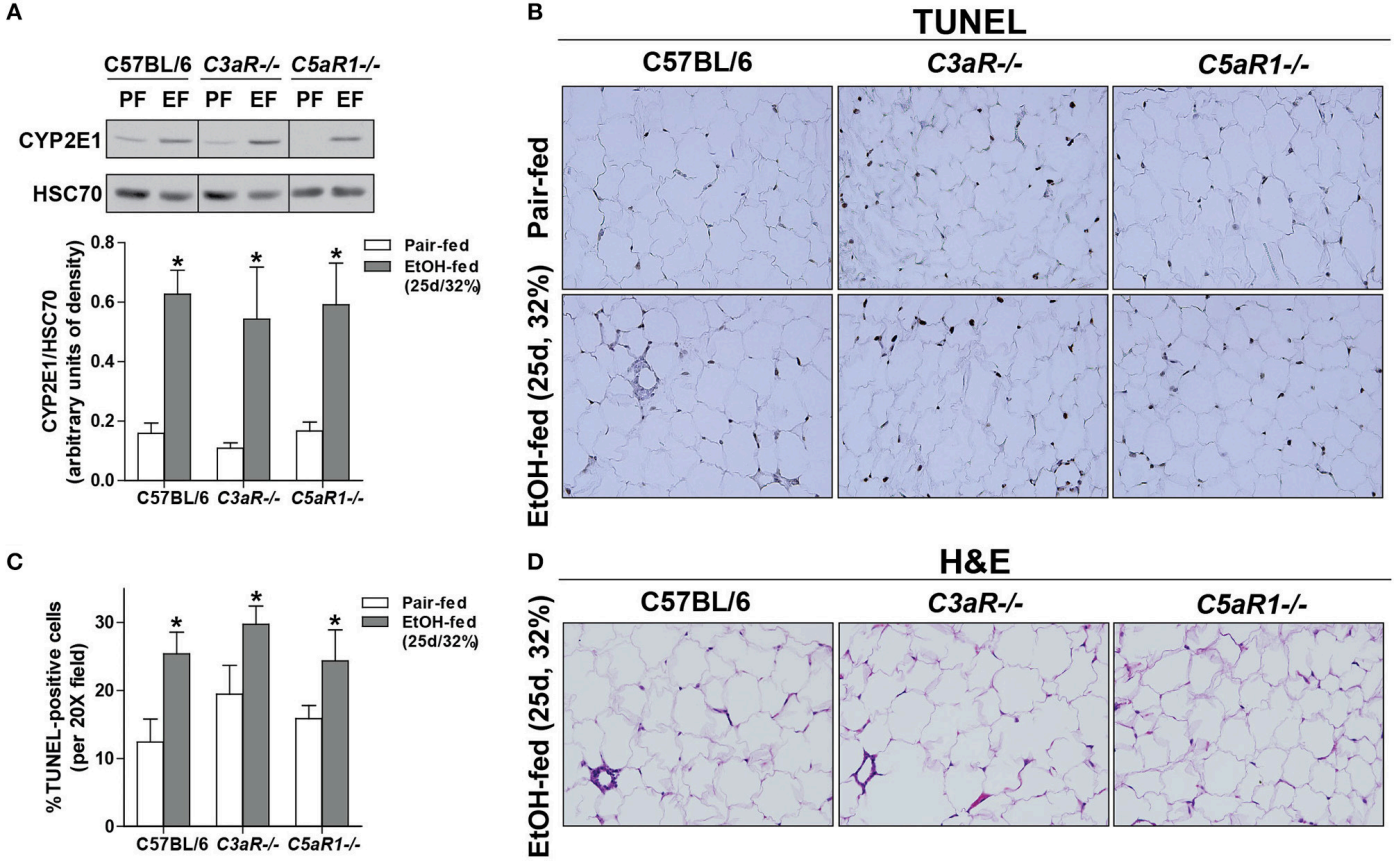
Chronic ethanol feeding leads to CYP2E1 induction and accumulation of TUNEL-positive cells in gonadal adipose tissue. WT, *C3aR*^−/−^, and *C5aR1*^−/−^ mice were allowed free access to ethanol (32%, d25) or pair-fed control diets. **(A)** Adipose lysates were prepared and proteins separated by SDS-PAGE. CYP2E1 and HSC70 (loading control) were measured by Western blot. Relative expression is denoted as arbitrary units of density. **(B)** Apoptotic cells were visualized in paraffin-embedded gonadal adipose sections. **(C)** TUNEL-positive cells were enumerated and expressed as a percent of the total hematoxylin-positive nuclei/20x field. All images were quantified from at least three images per slide. **(D)** Paraffin-embedded gonadal adipose sections were deparaffinized followed by staining with hematoxylin and eosin. Values represent means ± SEM, values with * were significantly different from pair-fed controls within genotype (*P*<*0.05*). *n* = 4–8 pair-fed and 6–8 EtOH-fed mice.

Oxidative stress is a well-described consequence of ethanol feeding in mice that contributes to cellular injury. Induction of CYP2E1, the major ethanol metabolizing enzyme, occurs in adipocytes from ethanol-fed mice (6). The expression of CYP2E1 protein was increased in gonadal adipose tissue of WT, *C3aR*^−/−^, and *C5aR1*^−/−^ mice after chronic ethanol feeding, with no differences between genotypes (Figure 2A).

Apoptosis is driven, at least in part, by increased inflammatory mediators and oxidative stress. Given that *C5aR1*^−/−^ mice are protected from ethanol-induced increases in pro-inflammatory mediators, but still have CYP2E1 induction in the adipose tissue, we hypothesized that chronic ethanol feeding could lead to C5aR1-dependent apoptosis and thus contribute to enhanced adipose inflammation. Chronic ethanol feeding increased the accumulation of TUNEL-positive cells in the gonadal adipose tissue in WT, *C3aR*^−/−^, and *C5aR1*^−/−^ mice (Figures 2B,C). Despite accumulating TUNEL-positive cells, crown-like structures were absent in *C5aR1*^−/−^ mice (Figure 2D). Taken together, chronic ethanol feeding leads to the induction of CYP2E1 and accumulation of TUNEL-positive cells in adipose tissue, independent of the anaphylatoxin receptors.

### Ethanol feeding increased the expression of anaphylatoxin receptors on isolated adipocytes and stromal vascular cells (SVCs)

**Figure 3:**
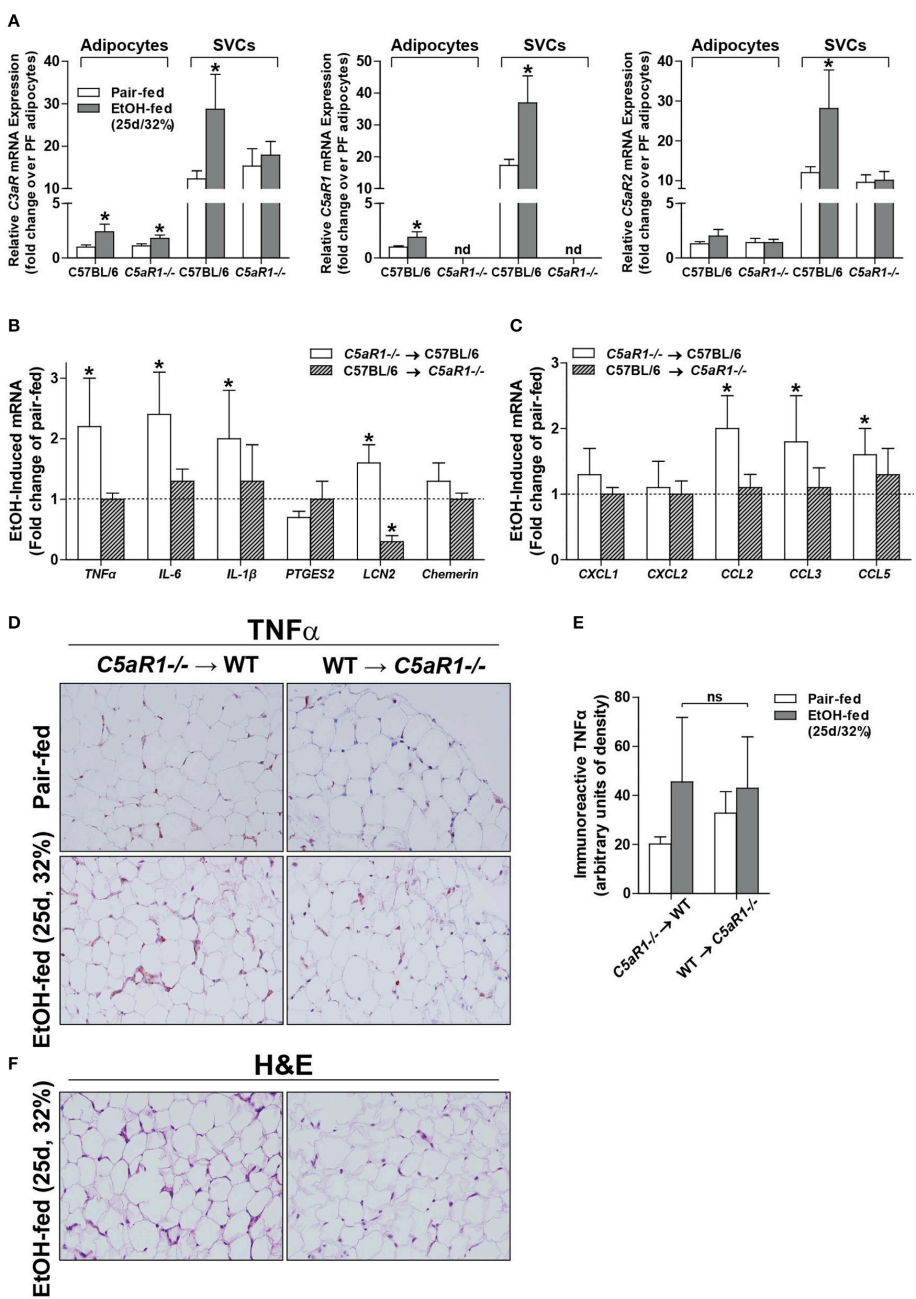
Non-myeloid C5aR1 expression modulates ethanol-induced adipokine expression in gonadal adipose tissue after chronic ethanol feeding in mice. WT and *C5aR1*^−/−^ mice were allowed free access to ethanol (32%, d25) or pair-fed control diets. Adipocytes and stromal vascular cells (SVCs) were isolated following collagenase digestion. **(A)** Expression of mRNA for anaphylatoxin receptors in adipocytes and SVCs. Data are represented as fold change of genotype pair-fed adipocyte expression; nd, not detected. Values represent means ± SEM, values with * were significantly different from pair-fed controls within genotype of each respective cell type (*P*<*0.05*). *n* = 4–8 pair-fed and 6–12 EtOH-fed mice. **(B–E)** Chimeras were generated via transplantation of WT or *C5aR1*^−/−^ bone marrow into *C5aR1*^−/−^ and WT recipients, respectively. Chimeric mice were allowed free access to ethanol (32%, d25) or pair-fed control diets. Expression of mRNA for **(B)** adipokines, cytokines and **(C)** chemokines in gonadal adipose tissue. Values are expressed as the fold increase over genotype pair-fed controls. Values represent means ± SEM, values with * were significantly different from pair-fed controls within genotype (*P*<*0.05*). *n* = 3 for pair-fed and *n* = 4 EtOH-fed mice. **(D)** Paraffin-embedded gonadal adipose sections were deparaffinized followed by immunodetection of TNFα. Nuclei were counterstained with hematoxylin. **(E)** TNFα-stained areas were quantified from at least three images per slide using Image-Pro Plus software. Values represent means ± SEM, ns = not significant between ethanol-fed groups. *N* = 3 for pair-fed and EtOH-fed mice. **(F)** Paraffin embedded gonadal adipose sections were deparaffinized followed by staining for hematoxylin and eosin. All images (at least three images/slide) were acquired using a 20 × objective; images are representative of 3–4 mice/group.

Chronic ethanol feeding increases the expression of C3aR and C5aR1 in adipose (6), but the cell type contributing to ethanol-mediated induction of the anaphylatoxin receptors in adipose is not known. Ethanol feeding increased expression of *C3aR* and *C5aR1*, but not *C5aR2* mRNA, in isolated adipocytes from WT mice compared to pair-fed controls (Figure 3A). In isolated adipocytes from *C5aR1*^−/−^ mice, ethanol feeding increased *C3aR*, but not *C5L2*, mRNA expression. Interestingly, isolated SVCs expressed ~10-fold higher levels of anaphylatoxin receptors; expression was further increased after ethanol feeding in WT, but not *C5aR1*^−/−^ mice.

### Adipocytes are the primary source of C5aR1-mediated induction of adipokines

Because C5aR1 is expressed on adipocytes and immune cells [Figure 3A, (30)], we next made use of bone marrow chimeras to determine which cell types contribute to the pro-inflammatory environment induced by ethanol feeding. Ethanol increased the expression of *TNF*α, *IL-6, IL-1*β *and LCN2*, but not *PTGES2* and *chemerin* mRNA in *C5aR1*^−/−^ → WT mice compared to pair-fed controls (Figure 3B). In contrast, WT → *C5aR1*^−/−^ chimeric mice were protected from ethanol-induced increases in cytokines and adipokines (Figure 3B). Ethanol increased the expression C-C chemokines *CCL2, CCL3, CCL5*, but not C-X-C chemokines *CXCL1* and *CXCL2* in *C5aR1*^−/−^ → WT chimeras compared to pair-fed controls, while WT → *C5aR1*^−/−^ chimeric mice were protected (Figure 3C). These findings suggest that non-myeloid cells, and likely adipocytes, are the primary source of C5aR1-mediated induction of adipokines after ethanol feeding in mice.

Similar to the induction observed in WT mice (Figures 1E,F), ethanol increased the expression of TNFα in gonadal adipose tissue in *C5aR1*^−/−^ → WT chimeras that was localized within crown-like structures (Figures 3D,E). Punctate TNFα expression was observed in cells appearing to be SVCs in ethanol-fed WT → *C5aR1*^−/−^ chimeras. Consistent with lack of chemokine induction, crown-like structures were absent in WT → *C5aR1*^−/−^ mice (Figure 3F).

### C5aR1 is a potent regulator of the soluble secretome of isolated adipocytes after chronic ethanol feeding

**Figure 4:**
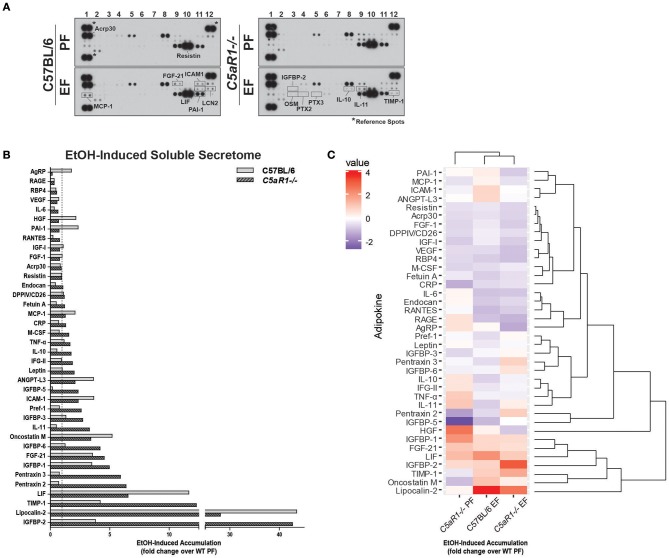
Ethanol and C5aR1 differentially modulates the soluble secretome from isolated adipocytes. WT and *C5aR1*^−/−^ mice were allowed free access to ethanol (32%, d25) or pair-fed control diets. Adipocytes were isolated following collagenase digestion. Spontaneous secretion of adipokines was assessed in supernatants 60 min post-isolation. **(A)** Adipokine accumulation was measured using an adipokine array. Relative density was calculated using Image J; data were normalized to reference spots for each experimental group. Lighter exposures were used for more abundant adipokines (Acrp30, Resistin) and reference spots (data not shown). **(B)** Ethanol-fed groups are expressed as fold change of WT pair-fed adipocyte supernatants. **(C)** Heat maps illustrate the changes in concentration for adipokines between groups; data are expressed as fold change of WT pair-fed adipocyte supernatants. Fold changes are shown in the column to the left of the heat maps (least abundant = dark blue; most abundant = red). Hierarchical clustering (shown at the right of the heat map) revealed the similarities between treatment and genotype. Data are representative of pooled samples (*n* = 6–8) for each group. PF, pair-fed; EF, EtOH-fed.

Given the importance of C5aR1 in regulating adipokine/cytokine production in adipose tissue, we hypothesized that C5aR1 would be an important contributor to ethanol-induced changes in the adipose secretome. Chronic ethanol feeding regulated both the quantity and distribution of adipokines secreted from adipocytes in a C5aR1-dependent mechanism (Figure 4). In pair-fed controls, absence of C5aR1 lead to increased accumulation of a variety of mediators compared to WT (Figure S1A). Chronic ethanol feeding induced a predominate release of pro-inflammatory adipokines from isolated adipocytes from WT mice; 13 adipokines were increased and included MCP-1, ICAM-1, Oncostatin M (OSM), FGF-21, LIF, and LCN2 (Figures 4A,B). Consistent with a pro-inflammatory shift in the secretome, ethanol reduced the secretion of ~12 adipokines from adipocytes, including well-known anti-inflammatory mediators IL-10 and IL-11. In contrast, adipocytes from ethanol-fed *C5aR1*^−/−^ mice predominately released anti-inflammatory adipokines; ~21 adipokines were increased with IL-10, IL-11, Pentraxin-2 (PTX2), Pentraxin-3 (PTX-3), TIMP-1, and IGFBP-2 being the most highly upregulated (Figures 4A,B). Interestingly, ethanol reduced the secretion of AgRP, a known negative regulator of leptin secretion (31), from ethanol-fed *C5aR1*^−/−^ adipocytes.

Hierarchical clustering analysis identified several adipokine clusters that were similarly affected by ethanol and C5aR1 (Figure 4C). Chronic ethanol feeding upregulated the PAI-1/MCP-1/ICAM-1/ANGPT-L3 and OSM/LCN-2 clusters and downregulated the PTX-3/IGFBP-6 and IL-10/IFG-II/IL-11 adipokine clusters in a C5aR1-dependent manner. Ethanol upregulated the IGFBP-2/TIMP-1 cluster, which was further increased in ethanol-fed *C5aR1*^−/−^ mice. Lastly, the Acrp30/Resistin/FGF-1 adipokine cluster was downregulated by ethanol, independent of genotype. One outlier, hepatocyte growth factor (HGF), was identified because ethanol feeding normalized higher HGF levels from pair-fed *C5aR1*^−/−^ supernatants. Taken together, ethanol feeding induced a pro-inflammatory shift in the adipocyte secretome that was C5aR1-dependent. Moreover, the adipocyte secretome from ethanol-fed *C5aR1*^−/−^ mice consisted of anti-inflammatory and protective adipokines, suggesting C5aR1 is a potent regulator of both pro- and anti-inflammatory factors from adipocytes in mALD.

### Ethanol-mediated secretion of adipokines/cytokines is C5aR1-dependent in isolated adipocytes

**Figure 5:**
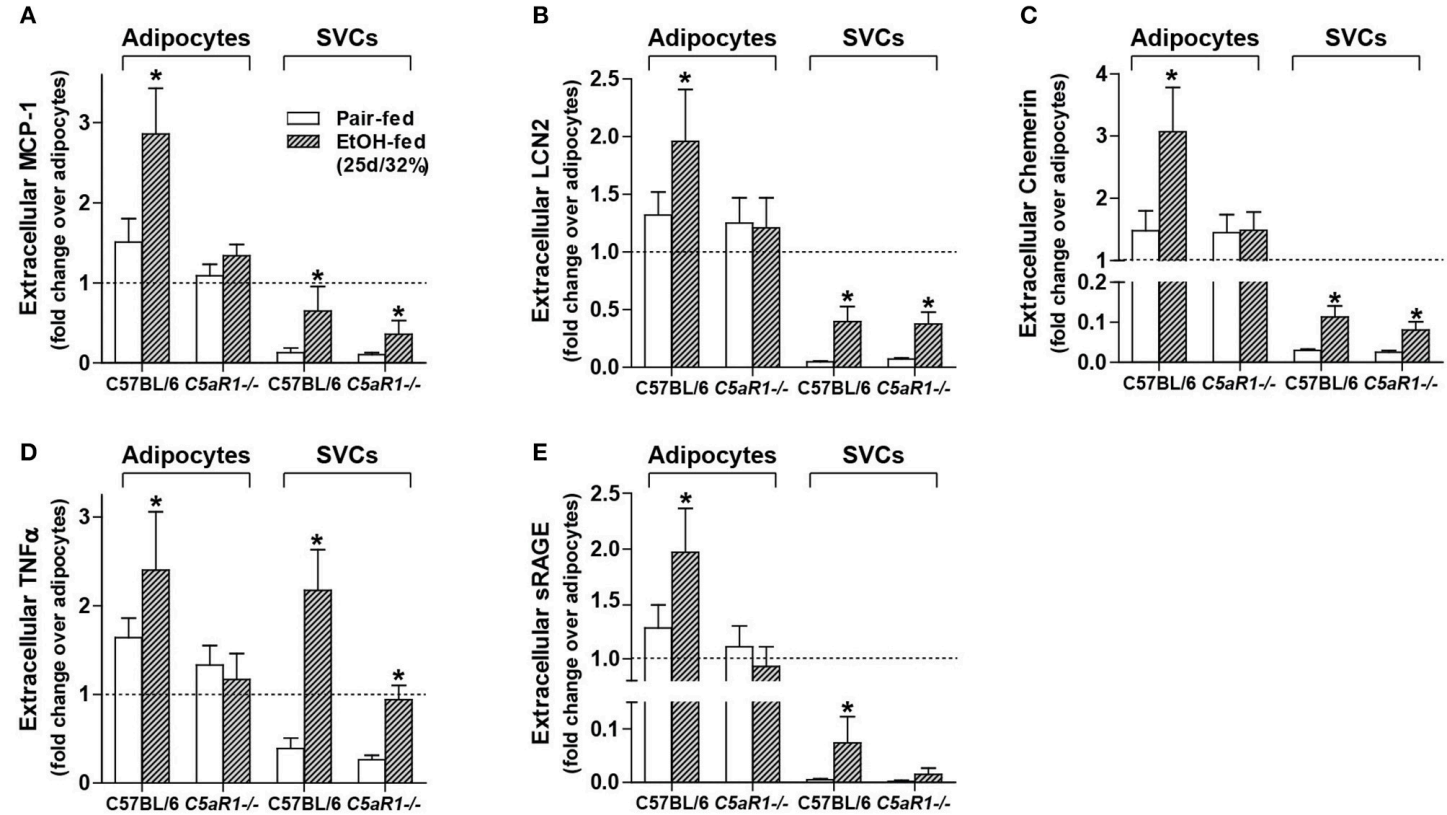
Ethanol-induced secretion of adipokines from isolated adipocytes, but not SVCs, is C5aR1-dependent. WT and *C5aR1*^−/−^ mice were allowed free access to ethanol (32%, d25) or pair-fed control diets. Adipocytes and stromal vascular cells (SVCs) were isolated following collagenase digestion. Spontaneous secretion of **(A)** MCP-1, **(B)** LCN2, **(C)** Chemerin, **(D)** TNFα, and **(E)** sRAGE was quantified from isolated adipocyte and SVC supernatants by ELISA. For adipocytes, data are represented as fold change accumulation after 30 min (LCN2, chemerin, sRAGE) or 60 min (MCP-1, TNFα) compared to the respective basal time zero of that group. SVC data are fold change accumulation after 60 min compared to adipocyte basal time zero within each respective genotype and treatment. Values represent means ± SEM, values with * were significantly different from genotype pair-fed controls (*P*<*0.05*). *n* = 6–8 pair-fed and *n* = 6–8 EtOH-fed mice.

To further investigate the role of C5aR1 on the modulation of the secretome, secretion of specific mediators were measured from isolated adipocytes and SVCs. Ethanol feeding increased the accumulation of MCP-1, LCN2, Chemerin, TNFα, and sRAGE in isolated adipocytes from WT, but not *C5aR1*^−/−^ mice compared to pair-fed controls (Figures 5A–E). Ethanol-induced accumulation of MCP-1, LCN2, chemerin and sRAGE from isolated SVCs was less compared to isolated adipocytes (Figures 5A–C,E). The accumulation of MCP-1, LCN2 and chemerin increased in SVCs from ethanol-fed WT and *C5aR1*^−/−^ mice (Figures 5A–C). sRAGE was increased in isolated SVCs from WT, but not *C5aR1*^−/−^ mice compared to pair-fed controls (Figure 5E).

Consistent with myeloid and non-myeloid cells producing TNFα (Figures 3D,E) and similar to the accumulation observed in isolated adipocytes from WT mice, ethanol feeding increased the accumulation of TNFα from isolated SVCs from WT and *C5aR1*^−/−^ mice; SVCs from *C5aR1*^−/−^ mice secreted less TNFα compared to SVCs from ethanol-fed WT mice (Figure 5D). Taken together, these data demonstrate that while adipocytes and SVCs actively secrete adipokines in response to ethanol feeding, adipocytes are a major source of multiple components of the secretome. Moreover, we find that ethanol-mediated secretion in adipocytes is C5aR1-dependent.

### Adipocyte-derived extracellular vesicles (EVs) contain unique adipokine signatures differing from the soluble secretome

**Figure 6:**
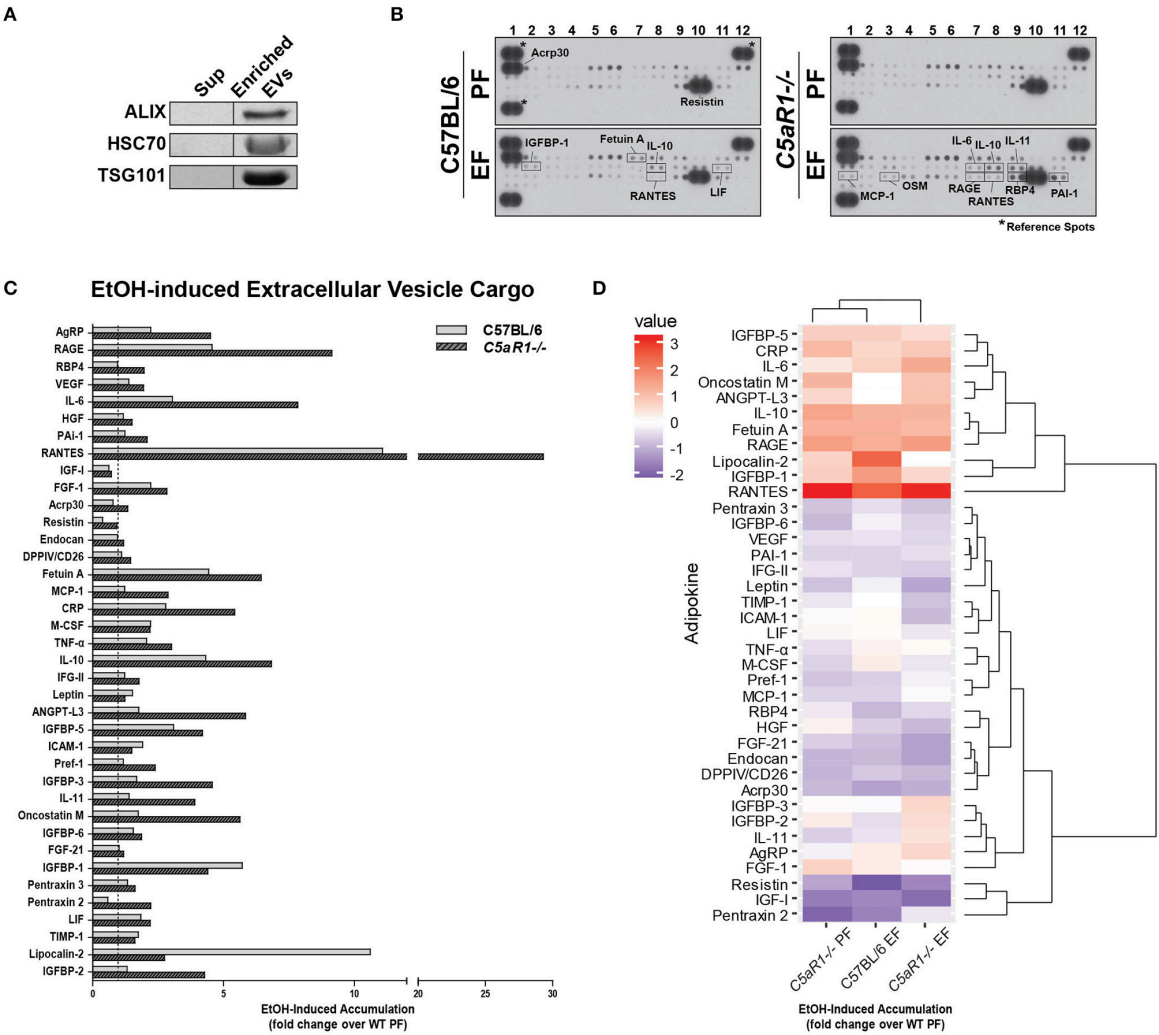
Ethanol and C5aR1 differentially modulate adipocyte-derived EV cargo. WT and *C5aR1*^−/−^ mice were allowed free access to ethanol (32%, d25) or pair-fed control diets. Adipocytes were isolated following collagenase digestion. Adipocyte-derived EVs were isolated from supernatants (120 min post-isolation) using PEG 8000-based precipitation. **(A)** EV content was prepared and proteins separated by SDS-PAGE. ALIX, HSC70, and TGS101 were measured by Western blot. **(B)** EV cargo was assessed using an adipokine array. Relative density was calculated using Image J; data were normalized to reference spots for each experimental group. Lighter exposures were used for more abundant adipokines (Acrp30, Resistin) and reference spots (data not shown). **(C)** Ethanol-fed groups are expressed as fold change of WT pair-fed EV cargo. **(D)** Heat maps illustrate changes in adipocyte-derived EV cargo between groups (least abundant = dark blue; most abundant = red); data are expressed as fold change of WT pair-fed EV cargo. Hierarchical clustering (shown at the right of the heat map) revealed the similarities between treatment and genotype. Data are representative of pooled samples (*n* = 6–8) for each group. PF, pair-fed; EF, ethanol-fed.

Extracellular vesicles (EVs) are important mediators of inflammation, involved in both intra- and inter-organ communication (2, 16). Because the specific cargo of EVs determine their physiologic/pathologic role, we next asked whether ethanol feeding modulated adipokine cargo in adipocyte-derived EVs and if C5aR1 was involved in this process. EV enrichment was confirmed by immunodetection of known EV markers ALIX, TSG101, and HSC70 (Figure 6A). Adipocyte-derived EV cargo was distinct from the soluble secretome (Figures 6B–D). Absence of C5aR1 changed adipokine cargo in adipocyte-derived EVs from pair-fed mice (Figure S1B). In WT EVs, ethanol increased the abundance of 20 adipokines, with the greatest induction of RANTES, Fetuin A, IGFBP-1 and LCN-2 (Figures 6B–D). EV cargo from ethanol-fed *C5aR1*^−/−^ adipocytes contained a greater diversity and higher concentration of adipokines; 30 adipokines were upregulated including sRAGE, IL-6, RANTES, IL-10, IL-11, and OSM.

Hierarchical clustering analysis revealed distinct differences between the secretome compartments (Figure 6D). Two large adipokine clusters, similarly affected by ethanol and C5aR1, were identified from adipocyte-derived EV cargo; one cluster was upregulated (red) while the other was downregulated (blue). Taken together, EVs contain a significant portion of adipokines that are modulated by both by ethanol and C5aR1.

## Discussion

Extrahepatic organs, including adipose tissue, are significant contributors to ALD progression. While exacerbated adipose inflammation has been reported in mALD (6, 32) and in ALD patients (33, 34), the mechanisms leading to this state are not well-understood. Here we report that chronic ethanol-induced adipose inflammation occurs in a C3aR- and C5aR1-dependent manner in mALD. Moreover, C5aR1 modulated the impact of chronic ethanol on the content of the adipose secretome as well as influencing adipokine cargo from adipocyte-derived EVs. These results identify for the first time the importance of complement in influencing the secretome that may impact organ-organ crosstalk in mALD.

Prolonged alcohol abuse induces chronic, low grade inflammation that fails to resolve, which in turn perpetuates tissue injury. Indeed, persistent activation or dysregulation of complement can also contribute to complement-mediated tissue damage in a number of chronic inflammatory diseases (35). Our data are the first to describe anaphylatoxin signaling as an important mediator in ethanol-induced adipose inflammation in mALD (Figure 1). Here we find that C5aR1 is a more potent regulator of adipose inflammation compared to C3aR, consistent with the potency of C5a compared to C3a in other cell types, including neutrophils and macrophages (36).

The similarities between *C3aR*^−/−^ and *C5aR1*^−/−^ mice in the response to ethanol is likely activation of similar downstream signaling modalities. For example, GPCR-mediated signaling of C3aR and C5aR1 stimulate multiple known pathways involved in ethanol-mediated pathologies, including cyclic adenosine monophosphate (cAMP), protein kinase B (AKT), forkhead box O1, nuclear factor kappa-light-chain-enhancer of activated B cells (NF-κβ), janus kinase 3/signal transducer and activator of transcription (JAK3/STAT3), and extracellular signal-regulated kinase 1/2 (ERK1/2) pathways (37, 38). Moreover, crosstalk between complement receptors and toll-like receptors are reported to enhance cell-specific functions, including cell activation, oxidative burst, and cytokine production (39, 40). While this relationship is not clearly addressed in our study, it will be important for future studies to more clearly define a possible relationship between C3aR/C5aR1 and TLRs in mALD.

The potency of C5aR1 on influencing the production of inflammatory factors in response to ethanol may be attributed to the ability of C5aR1 to homo- or hetero-dimerize with other innate immune components, including C5aR2 (41, 42) and chemokine receptors (43). This is further complicated by the cell type and stimuli associated with complement receptor activation; C5aR1 and C5aR2 co-localize on adipocytes, but not macrophages, following ASP or C5a stimulation (39). The physiologic function of C5aR2 remains enigmatic and controversial; as C5aR2 is a non-GPCR, initially it was thought to act as a decoy receptor (44). The role of C5aR2 in inflammatory diseases, and especially ALD, is not well-understood.

Ethanol metabolism via CYP2E1 increases the oxidative environment in adipose tissue and is an appreciable contributor to apoptotic cell death of adipocytes (6). Further, chronic ethanol-induced activation of complement is prevented in CYP2E1-deficient mice (6). Here we found that induction of CYP2E1 and adipocyte apoptosis after chronic ethanol feeding was independent of C3aR or C5aR1 expression (Figure 2). Taken together, these data indicate that ethanol metabolism via CYP2E1 is a critical initiator of complement activation and inflammation in adipose tissue.

Increased expression of chemokines like MCP-1 in adipose tissue leads to macrophage infiltration in models of diet induced-obesity and ethanol feeding in mice (45). Moreover, infiltrating macrophages surround dying adipocytes to form crown-like structures (46). The absence of crown-like structures in adipose from ethanol-fed *C5aR1*^−/−^ mice is likely due to the failure to upregulate the key chemokines that mediate this response (Figures 1, 2). Our data are consistent from Pheiler et al. who observed a similar protection from high-fat diet induced adipose inflammation and formation of crown-like structures in *C5aR1*^−/−^ mice (12).

The overall cellularity of adipose consists of adipocytes and the stromal vascular fraction, including perivascular cells, vascular endothelium, stem cells, pre-adipocytes and immune cells (1, 47). Because ethanol activates complement to generate anaphylatoxins (8, 18), known activators of the innate immune system, we hypothesized that adipose SVCs, including macrophages, would be the major contributor to ethanol-induced adipose inflammation in mALD. Despite high C5aR1 expression on SVCs compared to adipocytes, data from bone marrow chimeras suggest that C5aR1 expression on cells of non-myeloid origin, likely adipocytes, are the major contributor to the induction of inflammatory responses caused from ethanol feeding in adipose (Figure 3). In contrast, myeloid C5aR1 is the culprit for ethanol-induced hepatic inflammation (22). Taken together, the differential role of C5aR1 on multiple cells/organs may further complicate therapeutic targeting strategies in ALD.

The “secretome,” comprised of soluble mediators secreted from the adipose tissue, is an important homeostatic compartment regulating whole body metabolism, insulin sensitivity and inflammation. C5a-stimulated 3T3-L1 adipocytes secrete adipokines, including omentin, vaspin and chemerin, which is further enhanced in the presence of endotoxin (30). Moreover, C5a can stimulate chemokine release, including MCP-1 and CXCL1 in a C5aR-dependent manner (9). This is of particular interest for alcohol-mediated pathologies, as gut-derived LPS can stimulate multiple components of the innate immune system and perpetuate systemic inflammation. Indeed, peripheral C5a (8) as well as circulating endotoxin is increased in ALD patients (48, 49). Here we find that ethanol feeding, directly via the activation of complement, stimulates spontaneous secretion of multiple mediators, including chemokines (MCP-1 and chemerin), adipokines (LCN2) and pro-inflammatory cytokines (TNFα) from isolated adipocytes in a C5aR1-dependent manner (Figures 4, 5). Importantly, C5aR1 controlled the accumulation of multiple chemokines which are coordinately regulated into gene clusters (50). Because there are vast redundancies in the chemokine network (51), pharmalogical intervention targeting upstream factors, including complement, could perturb ethanol-mediated induction of entire chemokine networks in ALD.

C5aR1 also modulated the secretion of protective factors, including sRAGE, the peripheral sync for high-mobility group box 1 protein (HMGB-1), IL-10 and IL-11. These data are consistent with previous reports in HFD-induced obesity, as *C5aR1*^−/−^ mice have significant upregulation of IL-10 in the adipose (12). In addition, IGFBP-2, the mostly highly accumulated from *C5aR1*^−/−^ adipocytes, is associated with reduced susceptibility to obesity and improved insulin sensitivity (52), further supporting the injurious role of C5aR1 in adipose inflammation and dysfunction. Taken together, chronic ethanol feeding induced the spontaneous secretion of both pro-inflammatory and protective factors from isolated adipocytes in a C5aR1-dependent manner.

Important findings from our study revealed that 1) adipocyte-derived EVs contain a unique adipokine signature after ethanol feeding, 2) EV cargo differ vastly from the soluble secretome and 3) C5aR1 differentially modulates EV cargo after ethanol feeding. EVs, released in a highly regulated manner by activated or stressed cells, are broadly categorized as the compartment containing exosomes (< 100 nm) and microvesicles (>100 nm), respectively. While numerous cell types release EVs to the circulation to maintain homeostasis, modulation of their cargo, including non-coding RNAs, proteins and lipids, occurs in a number of inflammatory diseases, including ALD (15, 53). While little is known about their characteristics in adipose inflammation and mALD, ethanol feeding increased the content of numerous adipokines in adipocyte-derived EVs.

The interaction between C5aR1 and EVs is not well-understood, but some reports indicate C5aR1 may actually be packaged in neutrophil-derived EVs (54). Moreover, other complement components, including C5/C5a, are detected in EVs from ALD patients (55). We find that C5aR1 plays a role in determining the content of both pro-injury and pro-resolution adipokine cargo within adipocyte-derived EVs after chronic ethanol feeding. For example, in addition to containing high levels of RANTES, MCP-1, and TNFα, higher levels of gp130 cytokines IL-6, IL-10, IL-11 and OSM were also identified in adipocyte-derived EVs from *C5aR1*^−/−^ mice (Figure 6). Importantly, the abundance of chemokine receptor-2 (CCR2) and 5 (CCR5) ligands was different in EVs compared to the soluble secretome. Since CCR2 and CCR5 contribute to hepatic inflammation and fibrosis (56, 57), the abundance of CCR2/5 ligands in both soluble and EV compartments should be assessed when considering the use of CCR2/5 antagonists for chronic liver diseases. Collectively, EVs represent an additional mechanism by which adipose contributes to inter- and intra-organ crosstalk in ALD. In order to identify potential therapeutic targets, it will be important for future studies to more clearly define the roles of specific components of the secretome, soluble and EV-laden alike, to better understand organ-organ crosstalk in ALD.

## Conclusion

These data demonstrate C5aR1 is an important nexus between ethanol and adipose inflammation in mALD. Further, we have identified a novel role for C5aR1 in modulating the impact of ethanol on the content of the adipose secretome, as well as influencing the adipokine cargo in adipocyte-derived EVs.

## Author contributions

RM, MRM, and LN participated in research design; RM, MRM, and KP conducted experiments; RM and AK performed data analysis; RM and LN wrote or contributed to the writing of the manuscript; MEM provided complement receptor-deficient mice.

### Conflict of interest statement

The authors declare that the research was conducted in the absence of any commercial or financial relationships that could be construed as a potential conflict of interest.

## Supplementary material

Figure S1 C5aR1 effects a broad array of adipokines in the soluble secretome and EV cargo from isolated adipocytes in pair-fed mice. WT and *C5aR1*^−/−^ mice were allowed free access to ethanol (32%, d25) or pair-fed control diets. Adipocytes were isolated following collagenase digestion. Spontaneous secretion of adipokines and extracellular vesicle (EV) cargo were assessed in supernatants 60 and 120 min after the isolation, respectively. **(A)** Adipokine accumulation was measured using an adipokine array. Data (pair-fed groups only) are expressed as fold change of WT pair-fed adipocyte supernatants. **(B)** Adipocyte-derived EVs were isolated from supernatants using PEG 8000-based precipitation. EV cargo was assessed using an adipokine array; data (pair-fed groups only) are expressed as a fold change of WT pair-fed EV cargo. Relative density was calculated using Image J; data were normalized to reference spots for each experimental group. Data are representative of pooled samples (n = 6–8) for each group.

## Abbreviations

Acrp30: adiponectin
ALD: alcohol-related liver disease
C1q, complement component 1: Q subcomponent
C3: complement protein 3
C3a: anaphylatoxin fragment of C3
C3aR: C3a receptor 1
C5a: anaphylatoxin fragment of C5
C5aR1: C5a receptor 1
C5aR2: C5a receptor 2
CCL2 (MCP-1): C-C motif chemokine ligand 2 (macrophage chemoattractant protein-1)
CCL3 (MIP-1α): C-C motif chemokine ligand 3
CCL5 (RANTES): C-C motif chemokine ligand 5
CD11b: integrin subunit alpha M
CD11c: integrin subunit alpha X
CXCL1 (KC): C-X-C motif chemokine ligand 1
CXCL2 (MIP2α): C-X-C motif chemokine ligand 2
CYP2E1: cytochrome P450 2E1
EV: extracellular vesicle
F4/80: adhesion G protein-coupled receptor E1
FGF-21: fibroblast growth factor-21
GPCR: G-protein coupled receptor
HSC70: heat shock cognate 70 kDa protein
ICAM-1: intercellular adhesion molecule 1
IL-1β: interleukin-1 beta
IL-6: interleukin-6
LCN2: lipocalin 2
mRNA: messenger RNA
LIF: leukemia inhibitory factor
Ly6C: lymphocyte antigen 6C2
Ly6G: lymphocyte antigen 6G6e
OSM: oncostatin M
PTX2: pentraxin-2
PTX3: pentraxin-3
PAI-1: plasminogen activator inhibitor-1
PTGES2: prostaglandin E synthase 2
TIMP1: tissue inhibitor of metalloproteinase 1
TNFα: tumor necrosis factor-α
TSG101: tumor susceptibility gene 101
TUNEL: terminal deoxynucleotidyl transferase–mediated deoxyuridine triphosphate nick-end labeling.
