# Supplementary figures and images for: Anaphylatoxin Receptors C3aR and C5aR1 Are Important Factors That Influence the Impact of Ethanol on the Adipose Secretome
Source: Front Immunol. 2018 Sep 20;9:2133. doi: 10.3389/fimmu.2018.02133 (PMC6158367; doi:10.3389/fimmu.2018.02133)

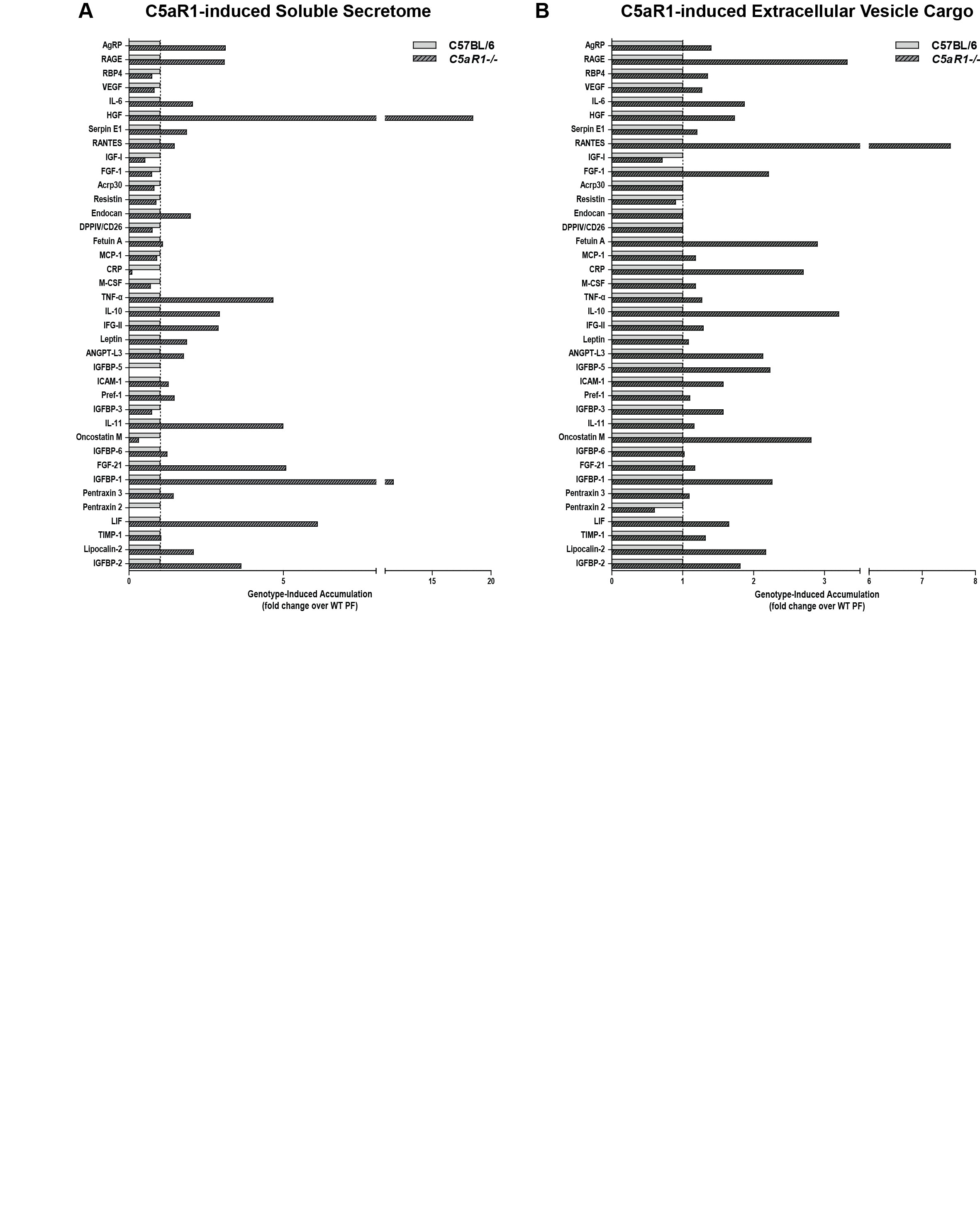

Supplement: Figure S1 — C5aR1 effects a broad array of adipokines in the soluble secretome and EV cargo from isolated adipocytes in pair-fed mice. WT and C5aR1−/− mice were allowed free access to ethanol (32%, d25) or pair-fed control diets. Adipocytes were isolated following collagenase digestion. Spontaneous secretion of adipokines and extracellular vesicle (EV) cargo were assessed in supernatants 60 and 120 min after the isolation, respectively. (A) Adipokine accumulation was measured using an adipokine array. Data (pair-fed groups only) are expressed as fold change of WT pair-fed adipocyte supernatants. (B) Adipocyte-derived EVs were isolated from supernatants using PEG 8000-based precipitation. EV cargo was assessed using an adipokine array; data (pair-fed groups only) are expressed as a fold change of WT pair-fed EV cargo. Relative density was calculated using Image J; data were normalized to reference spots for each experimental group. Data are representative of pooled samples (n = 6–8) for each group. [file Image_1.JPEG]
